# Epidemiology of Morbidity and Mortality of COVID-19 Patients During the Period of June 2020–September 2021 in Sulianti Saroso Infectious Disease Hospital, Indonesia

**DOI:** 10.21315/mjms2024.31.5.15

**Published:** 2024-10-08

**Authors:** Pompini Agustina Sitompul, Nina Mariana, Siti Maemun, Aninda Dinar Widiantari, Farida Murtiani, Rosamarlina Rosamarlina, Adria Rusli, Titi Sundari, Tri Bayu Purnama

**Affiliations:** 1 Pulmonology Department, Sulianti Saroso Infectious Disease Hospital, Jakarta, Indonesia; 2 Research Department, Sulianti Saroso Infectious Disease Hospital, Jakarta, Indonesia; 3 Faculty of Health Sciences, University of Respati Indonesia, Jakarta, Indonesia; 4 Division of International Health (Public Health), Graduate School of Medical and Dental Sciences, Niigata University, Niigata, Japan; 5 Faculty of Public Health, Universitas Islam Negeri Sumatera Utara, Medan, Indonesia

**Keywords:** COVID-19, hazard ratio, treatments

## Abstract

**Objectives:**

The increasing mortality rate of COVID-19 has remained an international public health concern. Limited studies on clinical treatment and morbidity in hospital settings are available in Indonesia. This present study aims to analyse demographic characteristics, clinical signs and treatment in COVID-19 patients and their association to the mortality case in Sulianti Saroso Infectious Disease Hospital.

**Methods:**

The study applied a retrospective cohort approach to all COVID-19 inpatients confirmed by polymerase chain reaction (PCR) testing in Sulianti Saroso Infectious Disease Hospital from 1 June 2020 to 30 September 2021. Overall survival rates until the end of the study were calculated using the Kaplan-Meier method and compared using the log-rank test. A Cox regression model was used to evaluate the crude and adjusted hazard ratios for associated factors.

**Results:**

We collected 1,970 inpatient data that met our inclusion and exclusion criteria. Most of them were 19 years old–59 years old (73.2%) and male (52.6%), and 966 (49%) patients had comorbidities. Approximately 63.9%, 89.2%, 89.8%, 82%, and 14.1% of the patients had ferritin levels ≤ 800, received antiviral treatment, were treated in non-intensive wards, had a moderate or mild clinical stage and did not survive, respectively. In the adjusted analysis, mortality was associated with sex (hazard ratio [HR]: 1.12; 95% CI: 1.02, 1.23), presence of comorbidity (HR: 1.19; 95% CI: 1.08, 1.30) and favipiravir (FPV) plus azithromycin treatment (HR: 1.21; 95% CI: 1.06,1.39). FPV treatment (HR: 1.35; 95% CI: 1.04, 1.75) was associated with higher mortality.

**Conclusion:**

Tailored approaches to treatment, considering individual risk factors and comorbidities, are crucial in improving patient outcomes.

## Introduction

COVID-19 has spread globally since 2020. Its transmission had been confirmed via droplet through coughing, sneezing and direct contact, which had proven to spread the respiratory disease rapidly. COVID-19 was first detected in Wuhan City, Hubei Province, China, in December 2019. In only a few months, it spread around Europe, the US and specific Asian regions (1, 2). Respiratory symptoms varied from mild to severe. Systemic inflammation, which occurs as a result of a cytokine storm, worsens patients’ condition, leading to sepsis and respiratory failure. Definite treatment for this viral infection has not been formulated.

Since the first identification of COVID-19 in Indonesia in March 2020, the country has experienced over 6.7 million documented cases and 160,956 deaths nationwide (3). Indonesia faced significant obstacles during the initial stages of the pandemic, including its expansive geography and insufficient laboratory infrastructure for COVID-19 diagnosis (4). Issues such as delays in contact tracing, difficulties in ensuring public compliance, and constraints on testing capacity further compounded the challenges faced by the country (5).

The predicted mortality rate was approximately 3.2% globally and the confirmed cases continued to rise (6, 7). Scientists around the world are still investigating who are considered the highest risk and the best treatment possible. We identify the etiology, risk factors, clinical signs, laboratory parameters, and management and treatment of COVID-19 in Sulianti Saroso Infectious Disease Hospital as one of the national referrals for COVID-19. The study aims to analyse the demographic characteristics, clinical signs, and treatment in COVID-19 patients and their association to the mortality case in Sulianti Saroso Infectious Disease Hospital.

## Methods

### Study Setting

This study was conducted in Jakarta, Indonesia from June 2020 to September 2021, amid the spread of the COVID-19 pandemic. During the study period, the location witnessed 2,395 cases by September 2021, as recorded in the Sulianti Saroso Infectious Disease Hospital, Jakarta, Indonesia, a public national referral hospital for infectious disease. It has experienced a major role in handling and preventing severe acute respiratory syndrome (SARS), H5N1, Middle East Respiratory Syndrome (MERS) and the COVID-19 pandemic.

### Study Design

**Figure 1:**
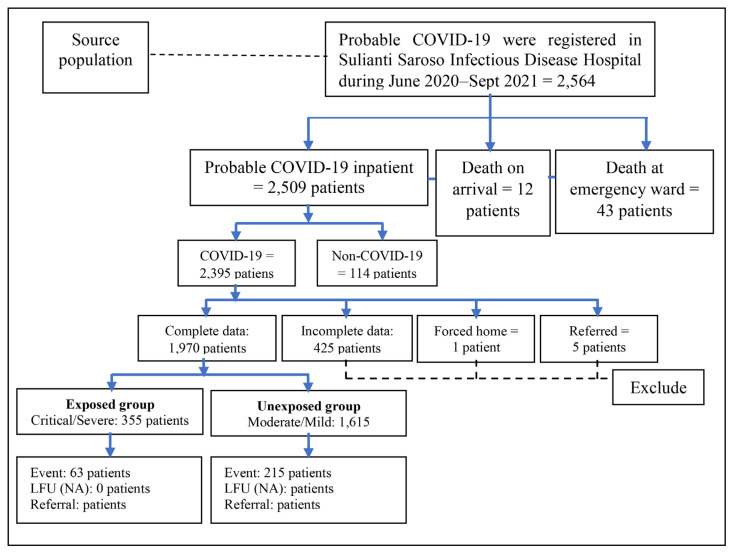
Data procedures

The COVID-19 data originated from Sulianti Saroso Infectious Disease Hospital during the study period. COVID-19 cases were confirmed through positive polymerase chain reaction (PCR) examination of laboratory specimens. This study compiled 2,395 inpatient COVID-19 cases and excluded 24 cases due to dead-on-arrival scenarios. A total of 1,970 inpatient COVID-19 cases were included in the final sample (Figure 1). The data collected from the hospital surveillance unit included demographic background, epidemiological characteristics, co-morbidity, clinical symptoms, severity, laboratory result, inpatient ward, length of stay (LoS) and clinical outcomes.

### Population and Sample

This study compiled 2,395 inpatient COVID-19 cases and excluded 24 cases due to dead-on-arrival scenarios. The study included patients who were hospitalised and tested positive for the Severe Acute Respiratory Infection Corona Virus (SARS-CoV-2) using real-time polymerase chain reaction (RT-PCR) assays. Patients with incomplete data or negative RT-PCR results for SARS-CoV-2 were excluded from the study. Through hypothesis testing for two population proportions at a 5% level of significance and 95% statistical power, we obtained 1,970 inpatient COVID-19 cases included in the final sample (Figure 1).

### Statistical Analysis

Normal distribution data were presented as minimum, maximum, mean and standard deviation (SD). Categorical variables in the analysis were shown in absolute numbers and their proportions (*n*, %). LoS between two independent groups was compared using a *t*-test. Overall survival is defined as the period from diagnosis time to death due to any cause or to the date of the last follow-up (LFU). Overall survival rates until the end of the study were calculated using the Kaplan-Meier method and compared using the log-rank test. Within the development cohort, all risk factors with a *P* < 0.05 in the univariable analysis were entered into the multivariable model to identify factors associated with overall survival. A Cox regression model was used to evaluate the crude and adjusted hazard ratios for associated factors.

We identified potential variables for the final prediction model based on *P*-values. When a parameter occurred in 60% or more of the bootstrap models, it was evaluated in the final multiple logistic regression model. Then, we computed the hazard ratios, 95% CIs and *P*-values for all metrics of the bootstrapped datasets in the final regression model. The final parameters used in the scoring system were defined by *P* = 0.05 in the final survival model. To confirm the risk score for each significant parameter, we adjusted the hazard ratio values. In the validation cohort, the area under the receiver operating characteristic (AUROC) curve was measured to evaluate the prediction accuracy of the survival rates after 14 days and 28 days. An AUROC value above 0.8 was considered reliable. Among the developed risk groups, we compared the LoS using one-way analysis of variance. For all statistical analyses, we used Stata 12 (Stata Corporation, College Station, TX, USA).

## Result

Confirmed COVID-19 cases were 19 years old–59 years old (73.2%), male (52.6%), Indonesian (97.9%) and live around Jakarta, Bogor, Depok, Tangerang and Bekasi. Few of them had a contact history (34.1%) and a traveling history (6.2%), but all of them had symptoms. Based on the kind of symptom, three symptoms had a general percentage greater than or equal to 25%, namely, fever (44.1%), cough (83.6%) and shortness of breath (44.9%). Other symptoms include rhinorrhea (19%), sore throat (10.6%), headache (19.9%), tiredness (22.1%), myalgia (2.1%), nausea/vomiting (21.7%), diarrhea (3.7%), anosmia (11.8%), ageusia (3.3%), loss of appetite (5.2%) and sore throat (1.8%).

Of the 1,970 patients, 49% (966) had comorbidities and 67.3% of them had one comorbidity. Two comorbidities had a general percentage ≥ 10%, namely, hypertension (28.1%) and diabetes mellitus (18.5%). A total of 3.2% and 0.6% of the patients acquired COVID-19 as a co-infection in pregnancy and a complication to acute respiratory distress syndrome (ARDS), respectively. Based on treatment, 1,757 (89.2%) patients received antiviral treatment, namely, favipiravir (FPV) (3.2%), remdesivir (6.1%), azithromycin (47.1%), azithromycin plus FPV (19.6%) and azithromycin plus remdesivir (13.2%). Of 1,970 patients, 57.3%, 89.8%, 82% and 14.1% were treated for at least 10 days, in non-intensive wards, had a moderate or mild clinical stage and did not survive, respectively (Table 1).

The Kaplan-Meier analysis showed significant differences in the survival curves between antiviral without azithromycin and none, received azithromycin and none, FPV plus azithromycin and none, LoS ≥ 10 days and < 10 days, obesity and not obesity, heart disease and none, and HIV/AIDS and none (log-rank test, *P* < 0.05). In the adjusted analysis (Table 2), mortality was associated with sex (HR: 1.12; 95% CI: 1.02, 1.23), the presence of comorbidities (HR: 1.19; 95% CI: 1.08, 1.30) and treatment of FPV plus azithromycin (HR: 1.21; 95% CI: 1.06, 1.39). FPV treatment (HR: 1.35; 95% CI: 1.04, 1.75) was associated with higher mortality. On the contrary, risk factors associated with lower mortality include treatment without azithromycin (HR: 0.88; 95% CI: 0.79, 0.98), ferritin levels ≤ 800 (HR: 0.84; 95% CI: 0.75, 0.94), comorbidity to bronchial asthma (HR: 0.68; 95% CI: 0.50, 0.92), obesity (HR: 0.12; 95% CI: 0.0, 0.47) and HIV/AIDS (HR: 0.56; 95% CI: 0.35, 0.89).

## Discussion

The study results revealed a 14.1% mortality rate of COVID-19, similar to a previous study held in Jakarta (8), whereas mortality rates of 4%–70% were reported globally (9–11). A study in a Peruvian referral hospital found that the mortality rate reached 46%. (12) In addition, a report from China explained that men showed greater risks to mortality rate (8, 13). As in several previous studies (7, 8, 14), the presence of comorbidity in our study was associated with increased mortality from COVID-19. Our analysis revealed that patients with one or two comorbidities had poor relation to the mortality, in contrast to research conducted in China (14).

This study highlighted that ferritin plays a significant role in predicting the mortality rate of COVID-19. A retrospective study conducted in Turkey showed that ferritin level was the only significant predictor of severity of COVID-19 (*P* = 0.004), with 88% AUC (15). In their study, Ahmed et al. (16) also found similar results where a significant difference in ferritin was observed between two severity groups. Binary logistic regression showed ferritin as an independent predictor of all causes of mortality with 69% AUC. A study in India also proved ferritin was correlated with clinical outcomes. Based on ROC analysis, the AUC score was 80.08% and the cut-off value was 352 ng/mL, with specificity and sensitivity of 76.32% and 74.5%, respectively (17). Ferritin levels increase during virus infection and can be the marker of its replication process. Increased ferritin is caused by cytokine storm and secondary haemophagocytic lymphohistiocytosis (sHLH), mainly in severe cases. Cytokine storm is marked by specific medical symptoms when excessive inflammation occurs in the body. Thus, it triggers multiorgan failure and mortality. During a cytokine storm, inflammatory cytokines are rapidly produced, including IL-6, TNF-α, IL-1β, IL-12 and IFN-γ, which sensitise the hepatocyte, Kupfer cell and macrophages to release ferritin. Ferritin serum is a trait of HLH, which is a complication of viral infection related to clinical deflation of COVID-19 patients. It is also known as the condition where the patient with worse lung lesion is more vulnerable to ferritin increase. A study stated that cytokine storm is mediated by pro-inflammatory cytokine, thus leading to acute lung injury and multiorgan failure (18). Mortality in severe COVID-19 cases was found to be correlated to cytokine storm initiated by inflammatory cytokines. However, clinical signs and biomarkers to predict the severity and mortality have not been found.

Our study showed that gender, comorbidities, treatment with FPV and treatment with FPV plus azithromycin were strong predictors of mortality, whereas treatment with azithromycin, ferritin levels, certain comorbidities, such as bronchial asthma, obesity, leukemia and HIV/AIDS, were poor predictors. These findings agree with the national data in July 2021, which recorded that 58% of COVID-19 cases were dominated by men. A study elsewhere found a similar finding, showing that most of the COVID-19 patients were men (67%) (19, 20). This vulnerability was correlated to their commuting to work; thus, the risk of exposure was higher than in women. In addition, cigarette and alcohol consumption weaken the immune system, thus increasing the risk of infection.

Having comorbidities was correlated to worse outcomes (21). Hence, people with comorbidities should be aware of this fact as the prognosis can be worse and even result in death (22). A UK Biobank study showed that asthma and non-allergic asthma had a higher risk of severe COVID-19 infection (23). Non-allergic asthma had more severe impacts on COVID-19 than atopic asthma. Potential mechanisms explain that atopic asthma is related to severe COVID-19 and leads to worse outcomes (24). Ramírez-Soto et al.’s (25) study suggests that as obesity prevalence increases, COVID-19 mortality rates increase in the Peruvian population aged ≥ 15 years old. These findings can help to elucidate the high COVID-19 mortality rates in Peru. Cohort study and meta-analysis results showed obesity was one of the independent variables related to severe COVID-19 and increased risk of mortality (21, 25). This association was explained by its connection to hypertension, diabetes and respiratory distress (26, 27). However, its direct mechanism for mortality and severe COVID-19 remains unrevealed. HIV caused twice the mortality risk compared with people with no HIV, considering demographic factors and lifestyles (28). A finding from Chanda et al. (29) also showed that in Zambia, uncontrolled HIV infection developed into severe cases more than the controlled one. Nevertheless, HIV infection was not independently related to worse outcomes among inpatient COVID-19 cases. Patients with chronic lymphocytic leukemia (CLL) are more vulnerable to the disease because of older average age and immunosuppression due to the treatment. A study reported that age, drug-related conditions and heart failure in CLL are significant risk factors for overall survival (30). Antileukemic treatment suppresses the immune system to exposed pathogens, thus increasing the susceptibility to infection (31). The impaired immune system of CLL inhibits the host from controlling the virus replication effectively. Thus, cytokine induces hyperactivated inflammation that causes acute respiratory distress syndrome, multiorgan impairment and death (32).

A randomised controlled trial in China that studied FPV efficacy compared with umifenovir reported no significant differences between the two drugs (33). Nevertheless, PCR conversion time in FPV was shorter (33). Less adverse effect was observed in FPV; thus, it is used as a COVID-19 drug in various countries. To obtain better outcomes, antivirus treatment should be started immediately after a diagnosis is made (34). However, in the Kaplan-Meier analysis comparing PCR conversion and survival rates, early treatment with FPV did not prove to alleviate survival. The reason for this occurrence may be due to various death-related factors. Azithromycin is indicated for the treatment of patients with mild to moderate infections caused by strains of sensitive microorganisms, such as upper respiratory infections (tonsillitis and pharyngitis) and lower respiratory infections (acute bacterial exacerbations, chronic obstructive pulmonary disease and community pneumonia). Single-dose azithromycin did not shorten conversion days compared with placebo. This finding did not support the routine use of the drug for COVID-19 outpatients (35). Azithromycin does not increase survival rate or other clinical conditions. The use of azithromycin for inpatient cases is limited to antimicrobial indications only (36).

## Conclusion

Tailored approaches to treatment, considering individual risk factors and comorbidities, are crucial in improving patient outcomes. Close monitoring of ferritin levels and timely interventions may aid in reducing mortality rates among COVID-19 patients. Continued surveillance and research are essential for refining treatment protocols and enhancing the overall management of COVID-19 cases.

## Figures and Tables

**Table 1:** Characteristics demography of COVID-19 patients

Variable	Severity		Outcome		Total

	Severe (*n* = 355) *N* (%)	Non-severe (*n* = 1,615) *N* (%)	Death (*n* =278) *N* (%)	Survived (*n* = 1,692) *N* (%)	
Sex					
Male	225 (63.4)	812 (50.3)	142 (51.1)	895 (52.9)	1037 (52.6)
Female	130 (36.6)	803 (49.7)	136 (48.9)	797 (47.1)	933 (47.4)
Age (years old)					
≥ 60 atau ≤ 18	131 (36.9)	396 (24.5)	78 (28.1)	449 (26.5)	527 (26.8)
19–59	224 (63.1)	1219 (75.5)	200 (71.9)	1243 (73.5)	1443 (73.2)
Nationality					
Foreign	0 (0)	42 (2.6)	8 (2.9)	34 (2.0)	42 (2.1)
Indonesian	355 (100)	1573 (97.4)	270 (97.1)	1658 (98.0)	1928 (97.9)
Domicile/Living					
Jakarta, Bogor, Depok Tangerang,	295 (83.1)	1339 (82.9)	230 (82.7)	1404 (83.0)	1634 (82.9)
Bekasi	60 (16.9)	276 (17.1)	48 (17.3)	288 (17.0)	336 (17.1)
Outside					
Referral					
Referral	205 (57.8)	252 (15.6)	74 (26.6)	383 (22.6)	457 (23.2)
Own visit	17 (4.8)	181 (11.2)	23 (8.3)	175 (10.3)	198 (10.1)
Other	127 (35.8)	1112 (68.9)	171 (61.5)	1068 (63.1)	1239 (62.9)
	6 (1.7)	70 (4.3)	10 (3.6)	66 (3.9)	76 (3.9)
Contact history					
Yes	56 (15.8)	615 (38.1)	92 (33.1)	579 (34.2)	671 (34.1)
None	299 (84.2)	1000 (61.9)	186 (66.9)	1113 (65.8)	1299 (65.9)
Travelling history					
Yes	9 (2.5)	114 (7.1)	22 (7.9)	101 (6.0)	123 (6.2)
None	346 (97.5)	1501 (92.9)	256 (92.1)	1591 (94.0)	1847 (93.8)
Fever					
Yes	163 (45.9)	706 (43.7)	130 (46.8)	739 (43.7)	869 (44.1)
None	192 (54.1)	909 (56.3)	148 (53.2)	953 (56.3)	1101 (55.9)
Cough					
Yes	281 (79.2)	1366 (84.6)	224 (80.6)	1423 (84.1)	1647 (83.6)
None	74 (20.8)	249 (15.4)	54 (19.4)	269 (15.9)	323 (16.4)
Rhinorrhea					
Yes	19 (5.4)	355 (22.0)	48 (17.3)	326 (19.3)	374 (19.0)
None	336 (94.6)	1260 (78.0)	230 (82.7)	1366 (80.7)	1596 (81.0)
Sore throat					
Yes	18 (5.1)	191 (11.8)	25 (9.0)	184 (10.9)	209 (10.6)
None	337 (94.9)	1424 (88.2)	253 (91.0)	1508 (89.1)	1761 (89.4)
Shortness of breath					
Yes	302 (85.1)	583 (36.1)	138 (49.6)	747 (44.1)	885 (44.9)
None	53 (14.9)	1032 (63.9)	140 (50.4)	945 (55.9)	1085 (55.1)
Headache					
Yes	39 (11.0)	354 (21.9)	52 (18.7)	341 (20.2)	393 (19.9)
None	316 (89.0)	1261 (78.1)	226 (81.3)	1351 (79.8)	1577 (80.1)
Tiredness					
Yes	84 (23.7)	351 (21.7)	65 (23.4)	370 (21.9)	435 (22.1)
None	271 (76.3)	1264 (78.3)	213 (76.6)	1322 (78.1)	1535 (77.9)
Myalgia					
Yes	4 (1.1)	38 (2.4)	5 (1.8)	37 (2.2)	42 (2.1)
None	351 (98.9)	1577 (97.6)	273 (98.2)	1655 (97.8)	1928 (97.9)
Diarrhea					
Yes	11 (3.1)	62 (3.8)	10 (3.6)	63 (3.7)	73 (3.7)
None	344 (96.9)	1553 (96.2)	268 (96.4)	1629 (96.3)	1897 (96.3)
Anosmia					
Yes	17 (4.8)	215 (13.3)	30 (10.8)	202 (11.9)	232 (11.8)
None	338 (95.2)	1400 (86.7)	248 (89.2)	1490 (88.1)	1738 (88.2)
Ageusia					
Yes	7 (2.0)	59 (3.7)	9 (3.2)	57 (3.4)	66 (3.3)
None	348 (98.0)	1556 (96.3)	269 (96.8)	1635 (96.6)	1904 (96.7)
No appetite					
Yes	18 (5.1)	84 (5.2)	12 (4.3)	90 (5.3)	102 (5.2)
None	337 (94.9)	1531 (94.8)	266 (95.7)	1602 (94.7)	1868 (94.8)
Myalgia					
Yes	5 (1.4)	48 (3.0)	1 (0.4)	52 (3.1)	53 (2.7)
None	350 (98.6)	1567 (97.0)	277 (99.6)	1640 (96.9)	1917 (97.3)
Sore chest					
Yes	4 (1.1)	32 (2.0)	6 (2.2)	30 (1.8)	36 (1.8)
None	351 (98.9)	1583 (98.0)	272 (97.8)	1662 (98.2)	1934 (98.1)
Precence of comorbidity					
Yes	241 (67.9)	725 (44.9)	149 (53.6)	817 (48.3)	966 (49.0)
None	114 (32.1)	890 (55.1)	129 (46.4)	875 (51.7)	1004 (51.0)
Amount of comorbidity (*n* = 966)					
5	1 (0.3)	0 (0)	0 (0)	1 (100)	1 (0.1)
4	6 (1.7)	4 (0.3)	2 (0.7)	8 (0.5)	10 (0.5)
3	12 (3.4)	31 (1.9)	2 (0.7)	41 (2.4)	43 (2.2)
2	64 (18.0)	198 (12.3)	39 (14.0)	223 (13.2)	262 (13.3)
1	158 (44.5)	492 (30.5)	106 (38.1)	544 (32.2)	650 (33.0)
Type of comorbidity					
Diabetes mellitus					
Yes	119 (33.5)	245 (15.2)	56 (20.1)	308 (18.2)	364 (18.5)
None	236 (66.5)	1370 (84.8)	222 (79.9)	1384 (81.8)	1606 (81.5)
Hypertension					
Yes	137 (38.6)	416 (25.8)	82 (29.5)	471 (27.8)	553 (28.1)
None	218 (61.4)	1199 (74.2)	196 (70.5)	1221 (72.2)	1417 (72.0)
Asthma bronchial					
Yes	5 (1.4)	40 (2.5)	5 (1.8)	40 (2.4)	45 (2.3)
None	350 (98.6)	1575 (97.5)	273 (98.2)	1652 (97.6)	1925 (97.7)
Heart disease					
Yes	11 (3.1)	58 (3.6)	5 (1.8)	64 (3.8)	69 (3.5)
None	344 (96.9)	1557 (96.4)	273 (98.2)	1628 (96.2)	1901 (96.4)
Cerebrovascular disease					
Yes	5 (1.4)	7 (0.4)	3 (1.1)	9 (0.5)	12 (0.6)
None	350 (98.6)	1608 (99.6)	275 (98.9)	1683 (99.5)	1958 (99.3)
Chronic kidney disease					
Yes	6 (1.7)	8 (0.5)	3 (1.1)	11 (0.7)	14 (0.7)
None	349 (98.3)	1607 (99.5)	275 (98.9)	1681 (99.3)	1956 (99.2)
Obesity					
Yes	8 (2.3)	6 (0.4)	5 (1.8)	9 (0.5)	14 (0.7)
No	347 (97.7)	1609 (99.6)	273 (98.2)	1683 (99.5)	1956 (99.2)
Leukemia					
Yes	2 (0.6)	0 (0)	0 (0)	2 (0.1)	2 (0.1)
No	353 (99.4)	1615 (100)	278 (100)	1690 (99.9)	1968 (99.9)
Chronic obstructive pulmonary disease					
Yes	1 (0.3)	3 (0.2)	0 (0)	4 (0.2)	4 (0.2)
No	354 (99.7)	1612 (99.8)	278 (100)	1688 (99.8)	1966 (99.8)
Cancer					
Yes	2 (0.6)	7 (0.4)	2 (0.7)	7 (0.4)	9 (0.5)
No	353 (99.4)	1608 (99.6)	276 (99.3)	1685 (99.6)	1961 (99.5)
Autoimmune					
Yes	1 (0.3)	4 (0.2)	1 (0.4)	4 (0.2)	5 (0.3)
No	354 (99.7)	1611 (99.8)	277 (99.6)	1688 (99.8)	1965 (99.7)
HIV/AIDS					
Yes	2 (0.6)	16 (1.0)	5 (1.8)	13 (0.8)	18 (1.0)
No	353 (99.4)	1599 (99.0)	273 (98.2)	1679 (99.2)	1952 (99.1)
Tuberculosis					
Yes	7 (2.0)	21 (1.3)	6 (2.2)	22 (1.3)	28 (1.4)
No	348 (98.0)	1594 (98.7)	272 (97.8)	1670 (98.7)	1942 (98.5)
Liver					
Yes	0 (0)	3 (0.2)	1 (0.4)	2 (0.1)	3 (0.2)
No	355 (18.0)	1612 (99.8)	277 (99.6)	1690 (99.9)	1967 (99.8)
Pregnancy					
Yes	5 (1.4)	59 (3.7)	9 (3.2)	55 (3.3)	64 (3.24)
No	350 (98.6)	1556 (96.3)	269 (96.8)	1637 (96.7)	1906 (96.8)
ARDS					
Yes	9 (2.5)	2 (0.1)	1 (0.4)	10 (0.6)	11 (0.6)
No	346 (97.5)	1613 (99.9)	277 (99.6)	1682 (99.4)	1959 (99.4)
Treatment					
Antiviral					
No	85 (23.9)	128 (7.9)	29 (10.4)	184 (10.9)	213 (10.8)
Yes	270 (76.1)	1487 (92.1)	249 (89.6)	1508 (89.1)	1757 (89.2)
Azitromicyn+Remdesivir					
No	283 (79.7)	1427 (88.4)	241 (86.7)	1469 (86.8)	1710 (87.0)
Yes	72 (20.3)	188 (11.6)	37 (13.3)	223 (13.2)	260 (13.2)
Azitromicyv+Favipiravir					
No	342 (96.3)	1241 (76.8)	225 (80.9)	1358 (80.3)	1583 (80.3)
Yes	13 (3.7)	374 (23.2)	53 (19.1)	334 (19.7)	387 (19.6)
Azitromicyn					
No	223 (62.8)	820 (50.8)	153 (55.0)	890 (52.6)	1043 (53.0)
Yes	132 (37.2)	795 (49.2)	125 (45.0)	802 (47.4)	927 (47.1)
Remdesivir					
No	309 (87.0)	1541 (95.4)	253 (91.0)	1597 (94.4)	1850 (94.0)
Yes	46 (13.0)	74 (4.6)	25 (9.0)	95 (5.6)	120 (6.1)
Favipiravir					
No	348 (98.0)	1559 (96.5)	269 (96.8)	1638 (96.8)	1907 (97.0)
Yes	7 (2.0)	56 (3.5)	9 (3.2)	54 (3.2)	63 (3.2)
Type of antiviral					
SoC	85 (23.9)	128 (7.9)	29 (10.4)	184 (10.9)	213 (11.0)
Favipiravir	7 (2.0)	56 (3.5)	9 (3.2)	54 (3.2)	63 (3.2)
Remdesivir	46 (13.0)	74 (4.6)	25 (9.0)	95 (5.6)	120 (6.1)
Azitromisin	132 (37.2)	795 (49.2)	125 (45.0)	802 (47.4)	927 (47.1)
Azitromisin and Favipiravir	13 (3.7)	374 (23.2)	53 (19.1)	334 (19.7)	387 (19.6)
Azitromisin and Remdesivir	72 (20.3)	188 (11.6)	37 (13.3)	223 (13.2)	260 (13.2)
Laboratory ferritin					
≤ 800	76 (21.4)	1182 (73.2)	169 (60.8)	1089 (64.4)	1258 (63.9)
> 800	279 (78.6)	433 (26.8)	109 (39.2)	603 (35.6)	712 (36.1)
Ferritin					
≤ 452.5	33 (9.3)	982 (60.8)	133 (47.8)	882 (52.1)	1015 (51.5)
> 452.5	322 (90.7)	633 (39.2)	145 (52.2)	810 (47.9)	955 (48.4)
LoS (days)					
≥ 10	223 (62.8)	906 (56.1)	159 (57.2)	970 (57.3)	1129 (57.3)
< 10	132 (37.2)	709 (43.9)	119 (42.8)	722 (42.7)	841 (4.7)
Ward					
Intensive	153 (43.1)	48 (2.9)	43 (15.5)	158 (9.3)	201 (10.2)
Non-intensive	202 (56.9)	1567 (97.0)	235 (84.5)	1534 (90.7)	1769 (89.8)
Outcome					
Death	63 (17.8)	215 (13.3)	–	–	278 (14.1)
Survived	292 (82.3)	1400 (86.7)			1692 (85.6)
Severity					
Severe	–	–	63 (22.7)	292 (17.3)	355 (18.0)
Non-severe			215 (77.3)	1400 (82.7)	1615 (82.0)

**Table 2:** Incidence rate ratio and Cox regression of the risk factors to death in COVID-19 patients

Variable	Log rank test	*P*-value	IRR	95% CI	*P*-value	cHR	95% CI	P-value	aHR	95% CI
Severity level	0.750	0.005	1.16	1.03, 1.31	0.000	1.33	1.18, 1.49	0.091	1.08	0.99, 1.17
Sex	0.069	0.024	1.10	1.00, 1.20	0.000	1.18	1.08, 1.28	0.019	1.12	1.02, 1.23
Age (year)	0.602	0.116	1.06	0.96, 1.18	0.028	1.12	1.01, 1.24			
Ward	0.231	0.067	1.17	0.96, 1.30	0.005	1.24	1.07, 1.43			
Comorbidity	0.997	0.006	1.12	1.02, 1.22	0.000	1.20	1.09, 1.31	0.000	1.19	1.08, 1.30
Antiretroviral and Azitro	0.611	0.456	0.99	0.86, 1.15	0.802	1.02	0.88, 1.17			
Antiretroviral without Azit	0.013	0.008	1.12	1.02, 1.22	0.000	1.20	1.10, 1.32			
Favipiravir	0.340	0.103	1.18	0.90, 1.51	0.019	1.35	1.05, 1.74	0.025	1.35	1.04, 1.75
Remdesivir	0.218	0.188	0.92	0.76, 1.11	0.099	0.86	0.71, 1.03			
Azitromicyn	0.039	0.008	0.90	0.82, 0.98	0.000	0.84	0.77, 0.92	0.015	0.88	0.79, 0.98
Favi+Azitro	0.045	0.000	1.21	1.08, 1.35	0.000	1.39	1.25, 1.56	0.005	1.21	1.06, 1.39
Remde+Azitro	0.950	0.430	0.99	0.86, 1.12	0.850	0.99	0.87, 1.13			
Ferritin 800	0.206	0.000	0.85	0.78, 0.94	0.000	0.76	0.70, 0.84	0.002	0.84	0.75, 0.94
Ferritin 452	0.202	0.000	0.85	0.78, 0.93	0.000	0.75	0.69, 0.82			
Cough	0.143	0.207	1.05	0.93, 1.18	0.442	1.05	0.93, 1.18			
Breathless	0.558	0.090	1.06	0.97, 1.16	0.020	1.11	1.02, 1.21			
Fever	0.325	0.374	1.01	0.93, 1.11	0.899	0.99	0.91, 1.09			
Fever breathless	0.292	0.028	1.12	1.00, 1.25	0.012	1.15	1.03, 1.29			
Cough breathless	0.523	0.137	1.05	0.96, 1.15	0.098	1.08	0.99, 1.18			
Cough fever	0.515	0.293	1.03	0.94, 1.13	0.837	1.01	0.92, 1.11			
Contact history	0.886	0.261	0.97	0.88, 1.07	0.349	0.96	0.87, 1.05			
Travel history	0.095	0.000	1.46	1.21, 1.76	0.000	1.83	1.52, 2.20			
Nationality	0.070	0.000	1.80	1.33, 2.51	0.000	2.43	1.79, 3.30			
Domicile	0.287	0.069	0.92	0.81, 1.03	0.007	0.85	0.76, 0.96			
LoS 10 days	0.000	0.000	2.44	2.23, 2.67	-	-	-			
Diabetes mellitus	0.547	0.033	1.11	0.99, 1.25	0.001	1.21	1.08, 1.36			
Hypertension	0.278	0.006	1.13	1.03, 1.25	0.000	1.23	1.11, 1.35			
Asthma bronchial	0.793	0.136	0.85	0.63, 1.16	0.034	0.73	0.54, 0.98	0.012	0.68	0.50, 0.92
Heart disease	0.043	0.200	1.11	0.82, 1.43	0.142	1.20	0.94, 1.52			
Cerebrovascular disease	0.180	0.353	0.91	0.52, 1.76	0.694	0.89	0.51, 1.57			
Kidney disease	0.905	0.150	1.32	0.76, 2.42	0.103	1.55	0.91, 2.62			
Obesity	0.000	0.129	0.73	0.44, 1.35	0.048	0.59	0.35, 0.99	0.019	0.53	0.31, 0.90
Leukimia	0.806	0.149	0.46	0.13, 3.77	0.015	0.18	0.04, 0.72	0.002	0.12	0.03, 0.47
Cronic obstructive disease	0.378	0.329	1.29	0.50, 4.73	0.439	1.47	0.55, 3.93			
Cancer	0.536	0.429	1.08	0.57, 2.37	0.768	1.10	0.57, 2.12			
Autoimun	0.496	0.365	0.88	0.38, 2.71	0.557	0.77	0.3, 1.85			
HIV/AIDS	0.007	0.163	0.79	0.50, 1.34	0.083	0.66	0.42, 1.05	0.015	0.56	0.35, 0.89
Tuberculosis	0.241	0.350	0.93	0.64, 1.41	0.599	0.90	0.62, 1.31			
Hepar disease	0.213	0.367	0.86	0.29, 4.17	0.694	0.80	0.26, 2.47			
Complication (ARDS)	0.365	0.390	1.10	0.62, 2.21	0.468	1.24	0.69, 2.26			
Pregnancy	0.638	0.363	1.05	0.82, 1.37	0.316	0.88	0.68, 1.13			
Amount of comorbidity (*n* = 966)										
5	–	–	–	–	0.460	0.48	0.07, 3.39			
4					0.634	0.86	0.46, 1.60			
3					0.062	0.75	0.55, 1.01			
2					0.002	0.80	0.70, 0.92			
1					0.003	0.86	0.78, 0.95			
None					Reference					

Notes: IRR = incidence rate ratio; HR = hazard rate; 95% CI = 95% confidence interval; *P*-value < α (0.05)
